# Analysis of Output Performance of a Novel Symmetrical T-Shaped Trapezoidal Micro Piezoelectric Energy Harvester Using a PZT-5H

**DOI:** 10.3390/mi13020282

**Published:** 2022-02-10

**Authors:** Wenda Xu, Hongrui Ao, Nannan Zhou, Zenghao Song, Hongyuan Jiang

**Affiliations:** Department of Machine Design, School of Mechatronics Engineering, Harbin Institute of Technology, Harbin 150001, China; xwd008169@163.com (W.X.); znn_hit1995@163.com (N.Z.); 17863107371@163.com (Z.S.); jhy_hit@hit.edu.cn (H.J.)

**Keywords:** micro piezoelectric energy harvester, T-shaped trapezoidal structure, output performance, substrate material

## Abstract

In recent years, low-power wireless sensors with high flexibility, portability and computing capability have been extensively applied in areas such as military, medicine and mechanical equipment condition monitoring. In this paper, a novel symmetrical T-shaped trapezoidal micro piezoelectric energy harvester (STTM-PEH) is proposed to supply energy for wireless sensors monitoring the vibrations of mechanical equipment. Firstly, the finite element model (FEM) of the STTM-PEH is established. Secondly, the modal analysis of the T-shaped trapezoidal piezoelectric cantilever beam is carried out by finite element software and its vibration modes are obtained. Additionally, the structural characteristics of the STTM-PEH and the composition of piezoelectric patches are described. Furthermore, the effects of resistance, acceleration coefficient, substrate materials and structural parameters of the output performance of the STTM-PEH are researched. The results indicate that the output power of the STTM-PEH rises first and then falls with a change in resistance, while the output voltage does not increase as resistance increases to a certain extent. Meanwhile, selecting copper as the piezoelectric material of the T-shaped trapezoidal piezoelectric cantilever beam can generate a higher energy output. Finally, how the structural parameters, including piezoelectric patch thickness, substrate thickness and cantilever head length, affect the output performance of the STTM-PEH is studied, which illustrates that the load range of the STTM-PEH can be appropriately broadened by adjusting the length of the cantilever beam head. This research is valuable for designing a novel high performance piezoelectric energy harvester.

## 1. Introduction

Recent decades have witnessed rapid innovation in micro-electro-mechanical systems (MEMS), system on chip (SoC) and microelectronic devices, which has promoted the widespread application of wireless sensors in areas such as medicine, the Internet of Things, agriculture, infrastructure monitoring and mechanical equipment condition monitoring [1,2,3,4,5,6]. Wireless sensors are conventionally powered by chemical batteries, but this method is facing severe challenges due to its defects such as environmental pollution, low energy storage density, restricted lifespan and high cost of replacement [7,8]. Hence, vibration energy harvesting technology is being extensively studied by researchers to solve the existing problem of a reliable power supply for wireless sensors. The energy harvested from the ambient vibrations can be converted into electric energy through electrostatic conversion, electromagnetic conversion, piezoelectric conversion, photoelectric conversion, and other methods [9,10,11]. Compared with other types of vibration energy harvesters, piezoelectric energy harvesters have the advantages of a high energy density, easy miniaturization and integration and no electromagnetic interference, which is very suitable for supplying power to wireless sensors [12,13,14].

The cantilever-type structure adopted by piezoelectric energy harvesters has the advantages of a simple structure, low resonance frequency and easy integration with micro-electro-mechanical systems [15]. To further enhance the piezoelectric efficiency of piezoelectric cantilever beams, the relevant researchers have developed multiform innovative work. X.M He proposed a novel MEMS piezoelectric vibration energy harvester based on trapezoidal cantilever beam array (TCBA-PVEH). The TCBA-PVEH has a smaller bending stiffness and larger piezoelectric strain energy per unit area than the rectangular cantilever beam array-based PVEH (RCBA-PVEH), thus giving it a lower resonance frequency and better electrical output [16]. Z.M. Wang proposed a flute-inspired mechanical-intelligent piezoelectric energy harvester to achieve self-tracking vibration frequency without manual intervention. This new energy harvester demonstrates a significant improvement of 610% in working bandwidth and an increase in power of 348% [17]. Z.B. Yang developed a high-efficiency compressive-mode piezoelectric energy harvester (HC-PEH). The HC-PEH shows a superior capability for high output power and favorable nonlinear phenomena at low frequency ranges [18]. Y.P. Wu designed a simple piezoelectric spring architecture based on a common binder clip structure. The proposed device could generate high output power at a low operating frequency [19].

On the other hand, the conventional piezoelectric energy harvesters based on linear mechanisms have the disadvantage of narrow bandwidth. Compared to linear mechanisms, nonlinearities provide rich dynamics such as sub- and super-harmonic resonances and in some cases chaotic behavior [20]. Thus, nonlinear techniques have been developed to improve the bandwidth of piezoelectric energy harvesters [21]. K.Y. Chen proposed and experimentally validated an auxetic nonlinear piezoelectric energy harvester [22]. This harvester adopts a clamp–clamp beam with auxetic structures, which can improve the efficiency and bandwidth of the energy harvesting based on a pure mechanical structure without outer magnets or stoppers. In the experimental validation, at 0.1 g base acceleration the power output of the two types of auxetic energy harvesters is 173% and 94% higher than the conventional nonlinear energy harvester. Moreover, the bandwidths of the two types of auxetic energy harvesters are broadened by 1556% and 2142% compared with linear systems. J.T. Zhang investigated a nonlinear distributed-parameter model for harvesting energy from galloping oscillation [23]. The stepped beam structure is beneficial for increasing the stress and strain of the piezoelectric elements, thereby enhancing power generation. The experimental work demonstrated that the optimal configuration of the energy harvester can improve the output power of the galloping phenomenon effectively.

In this paper, with the aim of synthesizing the structural characteristics of two traditional piezoelectric cantilever beams, T-shaped piezoelectric cantilever beams [24] and trapezoidal piezoelectric cantilever beams [25,26], we propose a novel T-trapezoidal piezoelectric energy harvester with a symmetrical structure (STTM-PEH). By reasonably adjusting the structural parameters of the STTM-PEH according to external conditions, a series of harvesters can be obtained and is able to capture ambient vibration with a frequency range of 5–35 Hz, which can provide steady energy for the wireless sensors monitoring the vibrations of mechanical equipment such as machine tools, 3D printers and electric machinery [27]. Firstly, PZT-5H was selected as the piezoelectric material of the STTM-PEH because of its higher brittleness, sensitivity, dielectric constant and electromechanical coupling coefficient than those of PVDF or AlN [28,29,30,31,32]. Subsequently, the finite element model of the T-shaped trapezoidal piezoelectric cantilever beam was established and its modal analysis was performed by COMSOL5.5 (COMSOL Inc, Stockholm, Sweden). Moreover, we explored the influence of external loading resistance and acceleration coefficient on the output performance of the STTM-PEH. Finally, the influence of the substrate material and geometric size of the T-shaped trapezoidal piezoelectric cantilever beam on the system were researched to optimize the output performance of the STTM-PEH compared with conventional piezoelectric energy harvesters. The low-carbon and environmentally friendly STTM-PEH proposed in this paper has high output performance, which can better convert ambient into electrical energy to supply power to wireless sensors. This research provides an innovative structure with high energy output for piezoelectric energy harvesters and has reference significance for realizing a continuous and reliable power supply for wireless sensors.

## 2. Structure and Method

### 2.1. Mechanism of Power Generation

Piezoelectric energy harvesters utilize the direct piezoelectric effect of the piezoelectric material to generate power [33,34]. When the piezoelectric material is deformed due to vibrations or pressure, polarized charges will be created on the upper and lower surfaces of it. There are two common coupling modes of direct piezoelectric effect: $d_{31}$ mode and $d_{33}$ mode [35]. The $d_{31}$ mode demonstrates that the direction of stress is perpendicular to the direction of the electrical field, while the $d_{33}$ mode shows that the direction of stress is parallel to the direction of the electrical field. The $d_{31}$ mode is generally utilized by cantilever-type piezoelectric energy harvesters to produce higher energy outputs.

Piezoelectric materials can produce electric displacement and electric fields under the action of stress and strain, and the quantitative relationship can be described by a piezoelectric equation. Two common constitutive piezoelectric equations are the strain–charge type and the stress–charge type, usually called d-type and e-type, respectively. These piezoelectric equations are as follows:

(1) $$\{\begin{array}{c} \delta =s\sigma +dE\;\;\;\;\;\;D=d\sigma +\varepsilon E\;\;\;\;\;\;\;\;d-type\\ \sigma =c\delta -eE\;\;\;\;\;\;D=e\delta +\varepsilon E\;\;\;\;\;\;\;\;\;e-type \end{array}$$

where D is electric displacement vector, E is electric field intensity, σ and δ represent stress and strain, respectively, d and c express the piezoelectric strain constant matrix and the elastic stiffness matrix, respectively, ε denotes relative permittivity, while s and e express the elastic flexibility coefficient matrix and the piezoelectric stress constant matrix, respectively. In this study, to improve the computational efficiency during the simulation process, the stress–charge constitutive piezoelectric equation is selected for the simulation.

### 2.2. Structure Design

The shape of the piezoelectric cantilever beam is one of the key factors affecting the output performance of the harvester [36,37]. Most piezoelectric cantilever beams are designed to be rectangular, but their stress distribution is uneven, which makes it difficult to efficiently utilize piezoelectric materials. In this study, combined with the characteristics of trapezoidal and T-shaped piezoelectric cantilever beams, a novel T-shaped trapezoidal micro piezoelectric energy harvester (STTM-PEH) with a symmetric structure is proposed. On the other hand, when designing the structural parameters of the T-shaped trapezoidal cantilever beam, the following factors should also be taken into account: Firstly, the harvester should be easy to install and economize space as much as possible without affecting the existing structure of the mechanical equipment. Secondly, the T-shaped trapezoidal cantilever beam ought to have a stable structure to ensure that the harvester will not be scrapped prematurely. Finally, the natural frequency of the STTM-PEH should be in the range of 5–35 Hz. Considering the above factors, the width of the trapezoidal cantilever body upper bottom is in the range of 11–15 mm and the width of its lower bottom is in the range of 36–51 mm. Moreover, the length range of the cantilever head is 10–16 mm, while the T-shaped trapezoidal cantilever beam length should not exceed 76 mm.

**Figure 1:**
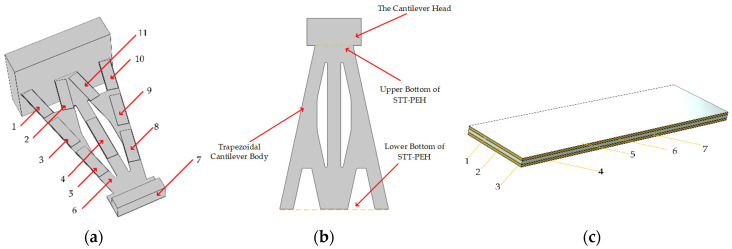
The structure of the STTM-PEH, T-shaped trapezoidal cantilever beam and piezoelectric patch. (**a**) 1,2,3,5,8,9,10,11—piezoelectric patches (piezoelectric layer: PZT-5H); 4—long piezoelectric patch (piezoelectric layer: PZT-5H); 6—T-shaped trapezoidal cantilever beam (copper); 7—mass (structural steel). (**b**) Structure of T-shaped trapezoidal cantilever beam. (**c**) 1,3—electrode; 2—piezoelectric material; 4,5—insulation layer; 6,7—conductive adhesive layer.

After the above analysis, the STTM-PEH is designed as shown in Figure 1. The T-shaped trapezoidal cantilever beam is composed of a cantilever head and a trapezoidal cantilever body as shown in Figure 1b. Table 1 displays the specific structural sizes of the T-shaped trapezoidal cantilever beam and the mass block. Additionally, in the design of piezoelectric materials, unlike a conventional piezoelectric energy harvester, we encapsulate the piezoelectric layer and electrode together using an insulation layer to make piezoelectric patches and install them in the stress concentration areas on the surface of the cantilever beam, which improves the effective utilization of the piezoelectric materials. The piezoelectric layer and electrode are bonded together with a conductive adhesive, and the different piezoelectric patches attached on the surface of the STTH-PEM are connected in parallel. Its structural details and components are shown in Figure 1c and Table 2.

### 2.3. Finite Element Model

**Figure 2:**
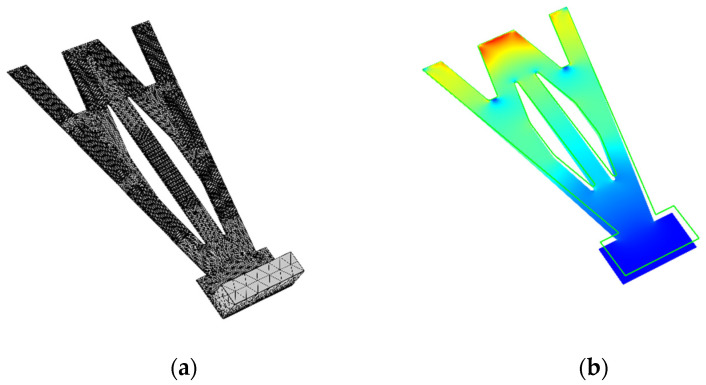
(**a**) meshes of the STTM-PEH; (**b**) stress distribution of T-shaped trapezoidal piezoelectric cantilever beam.

We use the finite element method (FEM, software COMSOL Multiphysics 5.5) to analyze the output performance of the STTM-PEH and the physical modules, selecting piezoelectric, circuit, solid mechanics and static electrostatic. Meanwhile, we use four-node free tetrahedron elements to generate meshes. To improve the computational efficiency, only the piezoelectric layer is retained in the simulation, while the conductive adhesive layer, electrode and packaging layer are ignored. Additionally, different refined meshes are applied to the cantilever beam, piezoelectric patches and mass to improve the calculation accuracy. The meshes of the STTM-PEH and stress distribution of the T-shaped trapezoidal piezoelectric cantilever beam are exhibited in Figure 2.

### 2.4. Modal Analysis

**Figure 3:**
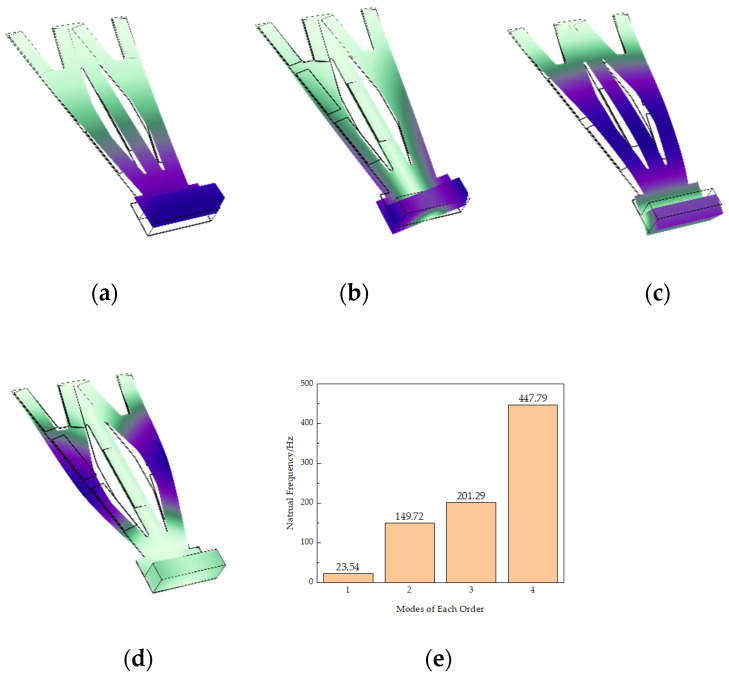
Various modes of the STTM-PEH (**a**) first-order mode; (**b**) second-order mode; (**c**) third-order mode; (**d**) fourth-order mode; (**e**) the natural frequencies of each mode of the STTM-PEH.

When the natural frequency of the piezoelectric energy harvester is consistent with the external vibration frequency, the energy collected by it reaches the maximum. Therefore, studying the modes of the STTM-PEH is beneficial for structural design and obtaining more outstanding output performance. Figure 3 shows various modes of the STTM-PEH. Figure 3a,b show the first-order and second-order modes of the harvester, while Figure 3c,d show the third-order and fourth-order modes, respectively. The natural frequencies of each mode are, respectively 23.54 Hz, 149.72 Hz, 201.29 Hz and 447.79 Hz, as show in Figure 3e.

The natural frequency of the STTM-PEH can be reduced to the specified range by mounting a mass block at the cantilever head. It is found that the cantilever beam mainly produces bending deformation in the first-order and third-order modes, while the torsion deformation mainly occurs in the second-order and fourth-order modes. The bending vibration of the first-order and third-order modes can generate greater energy in $d_{31}$ mode. In contrast, the second-order and fourth-order torsional vibrations produce less energy. In this paper, we focus on the piezoelectric output performance of the STTM-PEH under first-order natural frequency.

## 3. Results and Discussion

### 3.1. Influence of External Loading Resistance and Acceleration Coefficient on the Output Performance of the STTM-PEH

The external loading resistance has a great influence on the output performance of the piezoelectric energy harvester. Optimizing its resistance can allow the piezoelectric energy harvester to obtain maximum power. Therefore, the STTM-PEH as shown in Figure 1 is selected to study the variation of its output performance with the external loading resistance under acceleration of 1.0 g when the external excitation frequency is 23 Hz.

**Figure 4:**
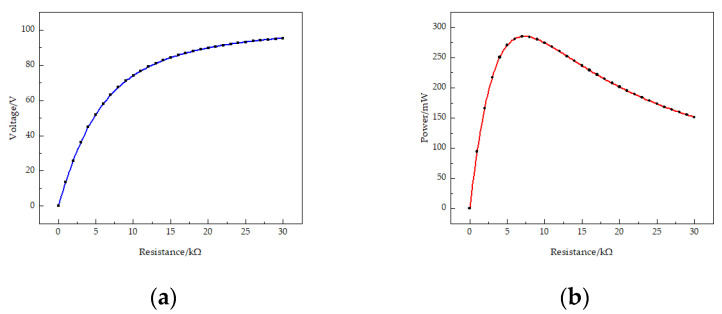

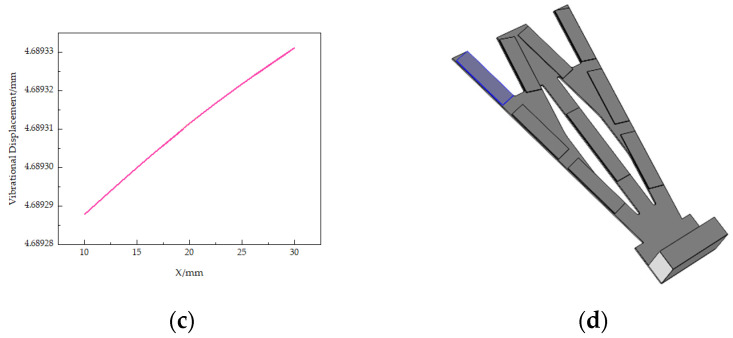
(**a**) Output voltage of the STTM-PEH with varying resistance; (**b**) output power of the STTM-PEH with varying resistance; (**c**) the vibrational displacement of each point at the tip of the beam; (**d**) the installation position of the piezoelectric patch with maximum stress on the T-shaped trapezoidal piezoelectric cantilever beam.

Firstly, the output voltage of the STTM-PEH is exhibited in Figure 4a as the resistance of external loading increases from 0 to 30 kΩ. The results illustrate that the output voltage rises with the loading resistance and tends to stabilize when the external loading resistance exceeds approximately 20 kΩ. The reason for this phenomenon is that when the resistance increases to a certain extent, the output side of the harvester is equivalent to an open circuit. At this time, increasing the resistance will not increase the output voltage. Secondly, Figure 4b shows the output power of the STTM-PEH with varying loading resistance, which shows that the output power of the harvester exhibits a rising trend at first before falling with an increase in resistance. When the resistance is about 7 kΩ, the output power of the harvester reaches its maximum, with an output power of about 284.8 mW and an output voltage of about 67.4 V. Thirdly, the vibration displacement of each point at the tip of the beam is shown in Figure 4c, where X represents the projection of the coordinates of each point at the tip of the beam on the x axis. Finally, it is found that the four piezoelectric patches installed near the root of the T-shaped trapezoidal piezoelectric cantilever beam bear greater stress. The maximum stress appears on the piezoelectric patch installed on the leftmost side of the beam root as shown in Figure 4d, which is about 126.2 GPa.

**Figure 5:**
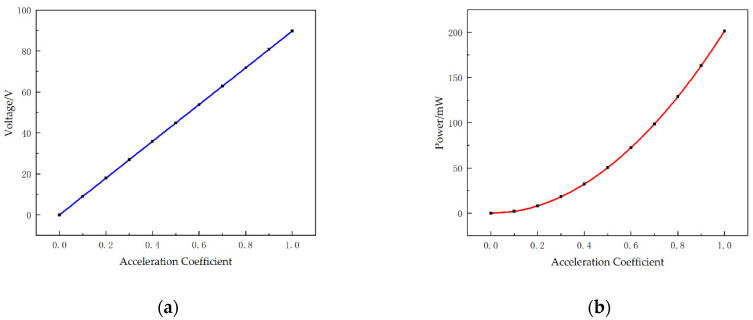
Output performance of the STTM-PEH with a varying acceleration coefficient. (**a**) Output voltage. (**b**) Output power.

On the other hand, the output performance of STTM-PEH is affected by external vibration excitation. Figure 5 shows the output voltage and output power of the STTM-PEH in the range of acceleration coefficients from 0 to 1.0 under the condition that the external loading resistance is 20 kΩ. Figure 5 indicates that the output voltage and power of the STTM-PEH increase as the acceleration coefficient rises. The output voltage of the STTM-PEH is proportional to the acceleration coefficient, while the growth rate of its output power–acceleration coefficient curve gradually increases. However, it should be noted that it is impossible to increase the acceleration coefficient indefinitely in practical applications.

### 3.2. Influence of T-Shped Trapezoidal Piezoelectric Cantilever Beam Substrate Material on the Output Performance of the STTM-PEH

When ambient vibration energy is harvested by the STTM-PEH, the stress and strain caused by vibrations will be transferred to the piezoelectric patches placed on the cantilever beam by the deformation of the T-shaped trapezoidal piezoelectric cantilever beam, which generates output voltage and power. Thus, reasonably selecting the substrate material of T-shaped trapezoidal piezoelectric cantilever beams an essential factor to ameliorate the output performance of the STTM-PEH. In this study, copper, chromium, silicon and structural steel are selected to explore the influence of different substrate materials on the output performance of the STTM-PEH when the structural parameters of the harvester are identical. The main physical parameters of copper, chromium, silicon and structural steel are exhibited in Table 3.

**Figure 6:**
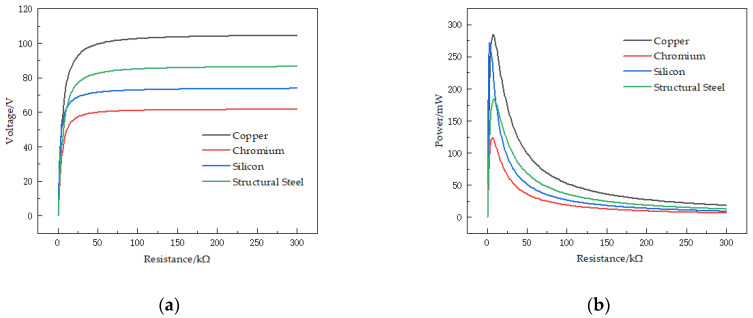
Output performance–resistance curves of the STTM-PEH under different substrate materials. (**a**) Output voltage–resistance curves. (**b**) Output power–resistance coefficient curves.

**Figure 7:**
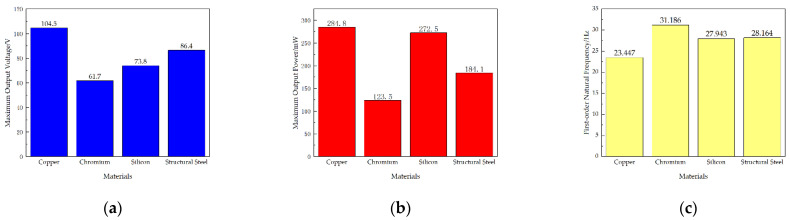
(**a**) Maximum output voltage of the STTM-PEH under different substrate materials. (**b**) Maximum output power of the STTM-PEH under different substrate materials. (**c**) First-order natural frequencies of the STTM-PEH under different substrate materials.

Firstly, the first-order natural frequencies of the STTM-PEH under four different substrate materials are obtained through modal analysis. Subsequently, under the condition that the acceleration coefficient is 1.0, the external excitations are applied to STTM-PEH under different substrate materials, and the excitation frequencies are 23 Hz (Copper), 31 Hz (Chromium), 27.3 Hz (Silicon) and 28 Hz (Structural steel), respectively. Figure 6 shows the output voltage–resistance curves and output power–resistance curves under different substrate materials. Furthermore, the relationships between the maximum output voltage and power of the STTM-PEH under different substrate materials are shown in Figure 7. The results illustrate that the highest maximum output voltage and power can be obtained when copper is selected as the substrate material of the T-shaped trapezoidal piezoelectric cantilever beam. Meanwhile, it was also discovered that Poisson’s ratio is positively correlated with the maximum output voltage, whereas Young’s modulus is inversely correlated with the maximum output power. The order of the Poisson ratios of these materials from high to low, as shown in Table 3, is copper (0.34) > structural steel (0.30) > silicon (0.28) > chromium (0.21), while the order of Young’s moduli of these materials is copper (120 GPa) < silicon (170 GPa) < structural steel (200 GPa) < chromium (279 GPa). Among these four materials, copper has the highest Poisson’s ratio and the lowest Young’s modulus, which can produce higher output voltage and power. Therefore, copper is selected as the substrate material for the T-shaped trapezoidal piezoelectric cantilever beam in this research.

### 3.3. Influence of Structural Parameters on the Output Performance of the STTM-PEH

In addition to choosing an appropriate substrate material for the T-shaped trapezoidal piezoelectric cantilever beam, optimizing the structure of the T-shaped trapezoidal piezoelectric cantilever beam can also yield a more ideal output performance for the STTM-PEH. In this section, the influence of geometrical factors on the output performance of the STTM-PEH is researched under the condition that the copper is selected as the substrate material of the T-shaped trapezoidal piezoelectric cantilever beam.

#### 3.3.1. Influence of Changing the Piezoelectric Patches Thickness of STTM-PEH

Through FEM, the influence of the piezoelectric patch thickness on the output performance of the STTM-PEH is studied under the condition that the thickness of the T-shaped trapezoidal cantilever beam substrate remains 0.3 mm. A series of piezoelectric patches is set and their thicknesses are 0.1 mm, 0.2 mm, 0.3 mm, 0.4 mm and 0.5 mm. Here, the variable r is introduced to characterize the ratio of piezoelectric patch thickness to T-shaped trapezoidal cantilever beam substrate thickness as shown in Equation (2):

(2) $$r=\frac{T_{p}}{T_{s}}$$

where $T_{p}$ represents the thickness of the piezoelectric patches and $T_{s}$ expresses the thickness of the T-shaped trapezoidal cantilever beam substrate.

**Figure 8:**
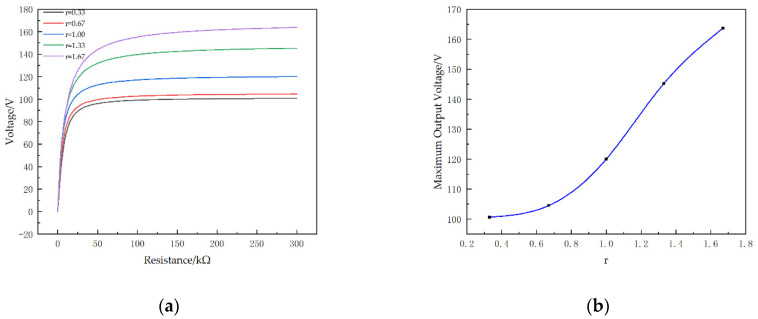
Output voltage of the STTM-PEH with different piezoelectric patch thicknesses. (**a**) Output voltage–resistance curves. (**b**) Maximum output voltage with different r.

The relationship between the resistance and output voltage of the STTM-PEH with different piezoelectric patch thicknesses is reflected in Figure 8 when the thickness of the substrate is constant. The result illustrates that when the resistance is greater than 50 kΩ the output voltage of the STTM-PEH tends to be stable and that its maximum output voltage increases with a rise in r. When the thickness of the piezoelectric patches is 0.5 mm (*r* is 1.67), the maximum output voltage is about 163.7 V.

**Figure 9:**
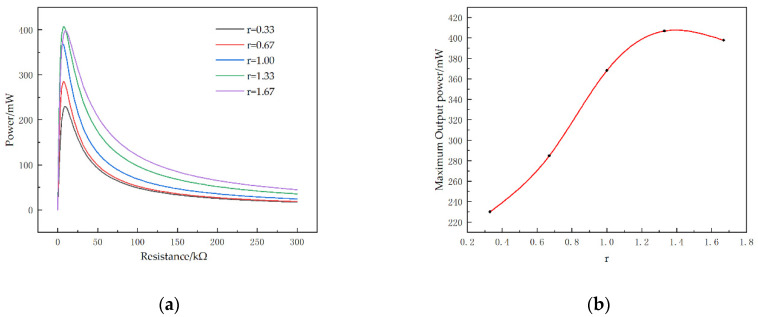
Output power of the STTM-PEH with different piezoelectric patch thicknesses. (**a**) Output power–resistance curves. (**b**) Maximum output power with different r.

Figure 9a shows that the output power–resistance curve falls more slowly with an increase in r. This means that the STTM-PEH with thicker piezoelectric patches can generate higher output power in the descent zone of its output power–resistance curve with identical external resistance. Hence, thicker piezoelectric patches should be selected for higher output power when the external resistance is greater than 50 kΩ. Moreover, in contrast to the variation in the maximum output voltage, the maximum output power increases first and then decreases with a rise in the piezoelectric patch thickness. When the thickness of piezoelectric patch is 0.4 mm (*r* is 1.33), the maximum output power of STTM-PEH is about 406.8 mW and its optimal resistance is about 8 kΩ, as shown in Figure 9b.

To conclude, it can be seen that increasing the thickness of the piezoelectric patches within a certain range can enhance the output performance of STTM-PEH, which is helpful for realizing higher output voltage and power. However, when the thickness of piezoelectric patches exceeds 0.4 mm, the maximum output power of the STTM-PEH will decrease. This is because excessively increasing the thickness of the piezoelectric patch raises the bending stiffness of the STTM-PEH, which reduces the deformation caused by external excitation, resulting in a decrease in the maximum power output.

#### 3.3.2. Influence of Changing the Thickness of the T-Shaped Trapezoidal Cantilever Beam Substrate

**Figure 10:**
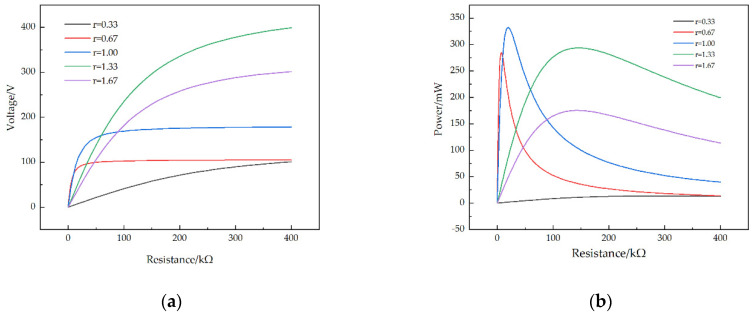
Output performance-resistance curves of the STTM-PEH with different T-shaped trapezoidal cantilever beam substrate thicknesses. (**a**) Output voltage-resistance curves. (**b**) Output power–resistance coefficient curves.

In addition to changing the thickness of the piezoelectric patches, changing the substrate thickness also affects the output performance of the STTM-PEH. By adjusting $T_{s}$, a group of STTM-PEHs are prepared under the condition of keeping the piezoelectric patch thickness of 0.2 mm unchanged for this experiment. The range of r is also 0.33–1.67, and the T-shaped trapezoidal cantilever beam thicknesses of these STTM-PEHs are 0.12 mm, 0.15 mm, 0.2 mm, 0.3 mm and 0.6 mm. Figure 10 presents the influence of different substrate thicknesses on the output voltage–resistance and output power–resistance curves and the relationship between the maximum output performance and the thickness of the substrate.

**Figure 11:**
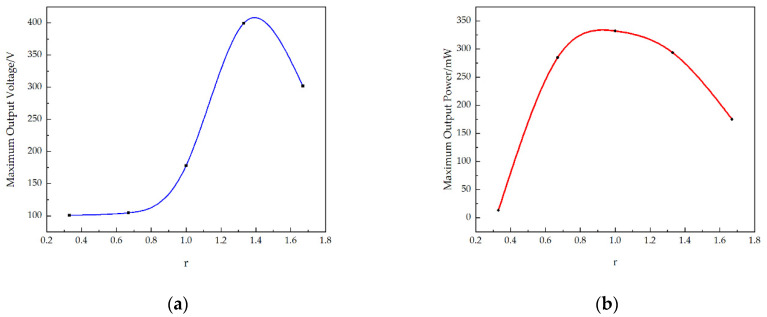
(**a**) Maximum output voltage of the STTM-PEH with different substrate thicknesses. (**b**) Maximum output power of the STTM-PEH with different substrate thicknesses.

Figure 10a and Figure 11a reveal that when r varies from 0.33 to 1.33 the maximum output voltage of the STTM-PEH increases, while when r varies from 1.33 to 1.67 the maximum output voltage of the STTM-PEH decreases. When the thickness of the T-shaped trapezoidal cantilever beam substrate is 0.15 mm (r is 1.33), the maximum output voltage that the STTM-PEH can achieve is the highest, which is about 399.4 V. Moreover, the relationship between the substrate thickness and the output power of the STTM-PEH is displayed in Figure 10b and Figure 11b, which indicate that the maximum output power of the STMM-PEH reaches its peak of 332.1 mW approximately when the substrate thickness is 0.2 mm (r is 1.00). In addition, it is found that the STMM-PEH with a substrate thickness of 0.12 mm and 0.15 mm can still generate output power above 100 mW as the external loading resistance increases from 200 kΩ to 400 kΩ.

In conclusion, when the T-shaped trapezoidal cantilever beam substrate thickness ranges from 0.3 mm to 0.12 mm, the STTM-PEH can produce greater output voltage and power. Meanwhile, a thinner substrate thickness of the T-shaped trapezoidal cantilever beam should be selected to produce higher output energy under the condition of high external loading resistance or open-circuit voltage. The main reason for the above findings is that the appropriate selection of a thin cantilever beam substrate can reduce the bending stiffness of STTM-PEH so that its vibration amplitude under the same external excitation increases and the stress near the piezoelectric patches rises, thus improving its output performance.

#### 3.3.3. Influence of Changing the Length of Cantilever Head

Besides the width of the upper and lower bottom, how the length of the cantilever head affects the output performance of the STTM-PEH was also studied. Here, we selected different STTM-PEHs with identical trapezoidal cantilever bodies and cantilever head lengths h of 10 mm, 12 mm, 14 mm and 16 mm.

**Figure 12:**
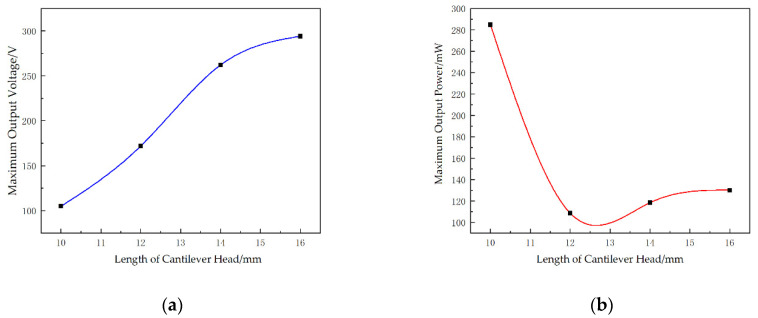
Maximum output performance of STTM-PEHs with different T-shaped trapezoidal cantilever head lengths. (**a**) Maximum output voltage. (**b**) Maximum output power.

It can be seen from Figure 12a that expanding the length of cantilever head can increase the maximum output voltage of the STTM-PEH. The maximum output voltage of 294.2 V is obtained with a STTM-PEH with a cantilever head length of 16 mm. However, unlike the maximum output voltage, the maximum output power decreases first and then increases with a rise in the cantilever head length, as shown in Figure 12b, which indicates that the optimal output performance cannot be obtained by simply increasing the length of the cantilever head.

**Figure 13:**
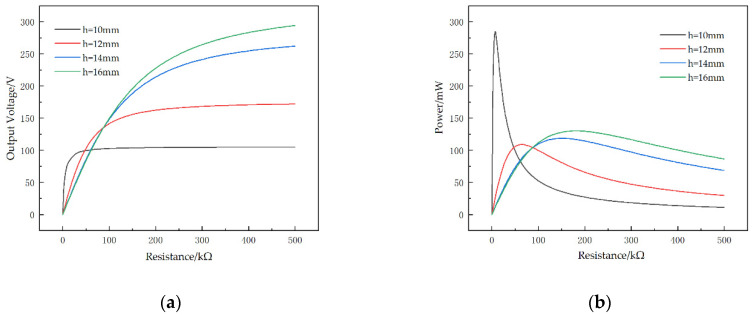
Output performance–resistance curves of the STTM-PEH with different lengths of the T-shaped trapezoidal cantilever head. (**a**) Output voltage–resistance curves. (**b**) Output power–resistance curves.

Figure 13 shows the voltage–resistance curves and the power–resistance curves for different cantilever head lengths. The results show that the shorter the cantilever head of the STTM-PEH, the faster the output voltage–resistance curve rises (Figure 13a). In addition, it can also be seen that the longer the length of the cantilever head, the slower the power–resistance curve falls in its descent zone (Figure 13b). Therefore, a STTM-PEH with a shorter cantilever head length is more suitable in a situation where a low external loading resistance is required, while a STTM-PEH with a longer cantilever head length should be selected when the external loading resistance exceeds 200 kΩ.

## 4. Conclusions

In this paper, a novel STTM-PEH that combines the structural characteristics of the T-shaped and trapezoidal piezoelectric cantilever beams is presented. A comprehensive finite element model is established for the proposed harvester. Furthermore, we study the modes of the STTM-PEH and the effects of the piezoelectric materials, resistance, acceleration coefficient and structural parameters of the T-trapezoidal piezoelectric cantilever beam on the output performance of the STTM-PEH.

(1) The various modes of the T-trapezoidal piezoelectric cantilever beam were obtained using COMSOL. The results illustrate that bending deformation is mainly produced in the first-order and third-order modes, while torsion deformation mainly occurs in the second-order and fourth-order modes. The greatest energy is generated by the STTM-PEH in the first-order mode.
(2) The output performance of the STTM-PEH is affected by the resistance and external excitation. At first, the output voltage of the harvester increases with an increase in resistance, but when the resistance increases to a certain extent, the voltage does not increase any more. Secondly, as the resistance increases, the output power of the STTM-PEH increases first and then decreases, which means that there is an optimal resistance for maximizing the output power of the harvester. Thirdly, the output voltage and power of the STTM-PEH increase with the acceleration coefficient.
(3) The output performance of the STTM-PEH with copper, chromium, silicon and structural steel as the substrate material of the T-shaped trapezoidal piezoelectric cantilever beam was studied. The results illustrate that a substrate material with a larger Poisson’s ratio can generate a higher output voltage and a material with a smaller Young’s modulus can produce higher output power. Among the selected substrate materials, copper generates the largest output voltage and power. Consequently, copper is utilized as the substrate material of the STTM-PEH to gain the highest possible power output.
(4) The effects of the structural parameters, including the piezoelectric patch thickness, the T-shaped trapezoidal cantilever beam substrate thickness and the cantilever head length, on the output performance of the STTM-PEH were comprehensively explored. The results illustrate that the STTM-PEH produces greater output voltage and power when the piezoelectric patch thickness ranges from 0.2 mm to 0.5 mm and the substrate thickness ranges from 0.15 mm to 0.3 mm. Meanwhile, a longer cantilever head length can obtain higher output power when the external loading resistance is more than 200 kΩ. Hence, we can optimize the output performance of the STTM-PEH by properly changing its geometric parameters.

## Figures and Tables

**Table 1:** Structural sizes of T-shaped trapezoidal cantilever beam and mass.

Heading	Size (mm)
Upper bottom of trapezoidal cantilever body	14
Lower bottom of trapezoidal cantilever body	40
Length of trapezoidal cantilever body	60
Cantilever head	20×10
Mass	20×5×6

**Table 2:** Structural sizes and parameters of piezoelectric patch.

Components of the STTM-PEH	Materials	Size (mm)	Piezoelectric Coefficient
Electrode	Aluminum	$15\times 4.5\times 10^{-4}$	-
Piezoelectric material	PZT-5H	15×4.5	−41 to 274
Insulating encapsulation layer	PET insulating sheet	$15\times 4.5\times 2.0\times 10^{-4}$	-
Conductive adhesive layer	Epoxy resin	$15\times 4.5\times 10^{-4}$	-

**Table 3:** Physical parameters of copper, chromium, silicon and structural steel.

Substrate Material	Young’s Modulus (GPa)	Density ($kg/cm^{3}$)	Poisson’s Ratio
Copper	120	8960	0.34
Chromium	279	7150	0.21
Silicon	170	2329	0.28
Structural steel	200	7850	0.30

## Data Availability

The data in the paper are in line with MDPI Research Data Policies.
